# Activation of AMPK Promotes Maturation of Cardiomyocytes Derived From Human Induced Pluripotent Stem Cells

**DOI:** 10.3389/fcell.2021.644667

**Published:** 2021-03-09

**Authors:** Liang Ye, Xinyuan Zhang, Qin Zhou, Bin Tan, Hao Xu, Qin Yi, Liang Yan, Min Xie, Yin Zhang, Jie Tian, Jing Zhu

**Affiliations:** ^1^Department of Pediatric Research Institute, Ministry of Education Key Laboratory of Child Development and Disorders, National Clinical Research Center for Child Health and Disorders (Chongqing), China International Science and Technology Cooperation Base of Child Development and Critical Disorders, Children’s Hospital of Chongqing Medical University, Chongqing, China; ^2^Chongqing Key Laboratory of Pediatrics, Chongqing, China; ^3^Department of Cardiovascular (Internal Medicine), Ministry of Education Key Laboratory of Child Development and Disorders, National Clinical Research Center for Child Health and Disorders (Chongqing), China International Science and Technology Cooperation Base of Child Development and Critical Disorders, Children’s Hospital of Chongqing Medical University, Chongqing, China; ^4^Department of Clinical Laboratory, Ministry of Education Key Laboratory of Child Development and Disorders, National Clinical Research Center for Child Health and Disorders (Chongqing), China International Science and Technology Cooperation Base of Child Development and Critical Disorders, Children’s Hospital of Chongqing Medical University, Chongqing, China

**Keywords:** hiPSCs, hiPSC-CMs, AMPK, mitochondrial, cardiomyocyte maturation

## Abstract

Human induced pluripotent stem cell (iPSC)-derived cardiomyocytes (CMs) (hiPSC-CMs) are a promising cell source for disease modeling, myocardial regeneration, and drug assessment. However, hiPSC-CMs have certain immature fetal CM-like properties that are different from the characteristics of adult CMs in several aspects, including cellular structure, mitochondrial function, and metabolism, thus limiting their applications. Adenosine 5‘-monophosphate (AMP)-activated protein kinase (AMPK) is an energy-sensing protein kinase involved in the regulation of fatty acid oxidation and mitochondrial biogenesis in cardiomyocytes. This study investigated the effects of AMPK on the maturation of hiPSC-CMs. Activation of AMPK in hiPSC-CMs significantly increased the expression of CM-specific markers and resulted in a more mature myocardial structure compared to that in the control cells. We found that activation of AMPK improved mitochondrial oxidative phosphorylation (OxPhos) and the oxygen consumption rate (OCR). Additionally, our data demonstrated that activation of AMPK increased mitochondrial fusion to promote the maturation of mitochondrial structure and function. Overall, activation of AMPK is an effective approach to promote hiPSC-CMs maturation, which may enhance the utility of hiPSC-CMs in clinical applications.

## Introduction

Cardiovascular disease (CVD) is the leading cause of human mortality worldwide, and 42% of deaths are caused by myocardial infarction and subsequent cardiac failure (Writing Group et al., 2016). However, current treatment strategies, including drug therapy and surgical interventions, cannot rescue necrotic cardiomyocytes, which lead to alterations in the size, shape, structure and function of the heart (Ibanez et al., 2018). Recent breakthroughs in the basic research and clinical application demonstrated the use of stem cells for the treatment of CVD (Nguyen et al., 2016; Yu et al., 2017). The development of induced pluripotent stem cells (iPSCs) provided possibilities to explore the mechanism. iPSCs are reprogrammed cells with features similar to the characteristics of embryonic stem cells, such as the capacity for self-renewal and differentiation into many cell types, including cardiomyocytes. And iPSCs include convenient sources, lack of immunological rejection, and compliance with ethical issues (Gnecchi et al., 2017; Yoshida and Yamanaka, 2017). Cardiomyocytes differentiated from human iPSCs (hiPSC-CMs) are promising tools for the development of drug screening, tissue engineering, and cardiac disease modeling platforms (Guan et al., 2014; Mummery, 2018). Although hiPSC-CMs share a variety of signature markers with endogenous cardiomyocytes, the degree of similarity of metabolic characteristics to those of mature mammalian cardiomyocytes is unknown. Various strategies can be used to successfully purify *in vitro* differentiated hiPSC-CMs; however, the cells have important differences from authentic adult human cardiomyocytes (Robertson et al., 2013; Musunuru et al., 2018). Adult cardiomyocytes are long and cylindrical, differentiated cells are more rounded and substantially shorter (Snir et al., 2003; Musunuru et al., 2018). Metabolism of hiPSC-CMs is characterized by fewer mitochondria and immature mitochondrial structures; thus, hiPSC-CMs largely depend on glycolysis rather than on β-oxidation of fatty acids for energy (Hattori et al., 2010; Kim et al., 2013). The gene expression profile of differentiated cells is quite distinct from that of adult cardiomyocytes with regard to calcium signaling and cardiac ion channel genes (Xu et al., 2009).

After somatic cells are reprogrammed into induced pluripotent stem cells, the cells need to improve glycolytic efficiency to maintain the pluripotent state. Therefore, glycolysis is increased, and aerobic respiration is decreased. This change in the metabolic mode is called “metabolic transformation” (Cliff and Dalton, 2017). On the other hand, some studies have shown that stem cells mainly provide energy through aerobic glycolysis to maintain stemness, and when stem cells undergo induction and differentiation into tissue-specific cells, the energy supply mode changes to oxidative phosphorylation (OxPhos) (Shum et al., 2016). hiPSCs express high levels of enzymes regulating glycolysis and have low activity of pyruvate dehydrogenase, which makes them produce ATP mainly through glycolysis to provide energy, while expression of the genes of enzymes related to the glycolysis pathway is downregulated in differentiated cells, and energy metabolism changes from glycolysis to OxPhos (Prigione et al., 2010; Varum et al., 2011; Zhang et al., 2016). hiPSCs do not rely on OxPhos to produce ATP and use aerobic glycolysis (Varum et al., 2011). When hiPSCs exit pluripotency, they undergo metabolic remodeling so that energy production is converted to a mechanism that heavily relies on OxPhos and less on glycolysis (Moussaieff et al., 2015). The results of an *in vitro* tracer uptake study indicated a shift in the use of metabolic substrate from glucose to fatty acids after cardiac differentiation, which was similar to that observed in naturally isolated human cardiomyocytes (Nose et al., 2018).

The metabolic activities of cardiomyocytes are quite complex. Under normal physiological conditions, various metabolic pathways are regulated to coordinate with each other to meet the energy demand of tissues and maintain the stability of the internal environment. This control can be achieved by regulation of the activity of enzymes, especially rate-limiting enzymes in metabolic pathways (Kolwicz et al., 2013). Adenosine monophosphate-activated protein kinase (AMPK) is an energy-sensing protein kinase that plays the key role in the regulation of cell energy metabolism (Herzig and Shaw, 2018). AMPK is an important regulator of cardiomyocyte energy homeostasis and is mainly used as a metabolic sensor to coordinate anabolic and catabolic activities in the cell by phosphorylation of various proteins involved in the metabolic pathways (Ke et al., 2018). In addition to the direct role of AMPK in the regulation of cardiomyocyte metabolism, AMPK can also directly or indirectly influence other cellular processes, such as the regulation of mitochondrial function, posttranslational acetylation, autophagy, mitochondrial autophagy, endoplasmic reticulum stress, and apoptosis (Arad et al., 2007; Lin and Hardie, 2018). Therefore, AMPK is involved in the control of various cellular processes that can influence the health and survival of cardiomyocytes. Studies have reported that the activation of AMPK increases fatty acid uptake and inhibits fatty acid synthesis by promoting fatty acid transport proteins in the cell membrane and increasing fatty acid β-oxidation (Arad et al., 2007). Sustained AMPK activation led to increased glucose and fatty acid uptake and an increase in the mRNA and protein expression of GLUT4 and CD36 concomitant to an increased in oxygen consumption rate (OCR) (Sarikhani et al., 2020). Previous studies demonstrated that fatty acid treatment of hPSC-CMs induced phosphorylation of AMPK, which ultimately increased cardiomyocyte maturation (Horikoshi et al., 2019; Yang et al., 2019). Therefore, regulation of AMPK to promote metabolic maturation is expected to promote the maturation of hiPSC-CMs.

In this study, we validated the hypothesis that AMPK promotes the metabolic maturation of hiPSC-CMs. In addition, we assessed whether the changes in AMPK activation influence cardiomyocyte mitochondrial function.

## Materials and Methods

### Human iPSC Culture and Cardiomyocyte Differentiation

Undifferentiated hiPSCs used in this study were obtained from Beijing Cellapy Biotechnology. hiPSCs were cultured in Matrigel (Corning, United States)-coated plates in feeder-free culture conditions with fresh PGM1 PSC culture medium (Cellapy, China), and the cells were passaged at 80–90% confluence using a dissociation solution (Cellapy, China). The schematic shown in Supplementary Figure S1A illustrates the cell culture, differentiation, and dissociation timeline. The undifferentiated hiPSCs were induced to differentiate into cardiomyocytes by transient activation/inhibition of the Wnt signaling pathway. Cardiac differentiation methods were adapted from previously published articles (Lian et al., 2012; Sharma et al., 2015). Cells at 90% confluence were used for differentiation (day 0), and the medium was replaced with RPMI 1640 (Sigma, United States) with B27 supplement minus insulin (Thermo Fisher Scientific, United States), which was considered basal medium. Day 0 cells were incubated with 6 μmol of CHIR99021 (GSK-3 inhibitor) (Selleck, United States) for 48 h (day 2). Day 2 cells were cultured in the basal medium for 24 h (day 3). Day 3 cells were treated with 5 μmol of IWP2 (WNT inhibitor) (Selleck, United States) for 48 h (day 5). Then, day 5 differentiated cells were cultured in the basal medium for 48 h (until day 7). On day 7, the medium was replaced by RPMI 1640 with B27 supplement with insulin (Thermo Fisher Scientific, United States). Approximately 8 days after differentiation, some cells began to beat (Supplementary Video 1). Subsequently, cells were maintained in the medium containing RPMI 1640 plus B27 supplement with insulin for 7 days followed by maintenance in the cardiac enrichment medium [RPMI 1640 medium (Thermo Fisher Scientific, United States) supplemented with 4 mM sodium L-lactate (Sigma-Aldrich, United States)]. Cells were maintained in the cardiac enrichment medium for 3 days. After this enrichment phase, the medium was changed to RPMI 1640 with knockout serum replacement (KSR) (Thermo Fisher Scientific, United States). On day 20, spontaneously beating cardiomyocytes were dissociated with TrypLE Express enzyme (Thermo Fisher Scientific, United States), centrifuged, resuspended, and replated onto Matrigel-coated plates for follow-up experiments. hiPSC-derived cardiomyocytes were treated with 0.5 mM AICAR (Selleck, United States) or vehicle (DMSO) for 7 days from day 23 to day 30 (Supplementary Video 2). All cultures were grown at 37°C in 5% O_2_ and 5% CO_2_.

### Quantitative Real-Time PCR and Mitochondrial DNA

Total RNA preparations were extracted with TRIzol reagent (TaKaRa, Japan) from the cells and quantified using a NanoDrop (Thermo Fisher Scientific, United States). One microgram of total RNA was reverse transcribed to cDNA using a PrimeScript RT reagent kit (TaKaRa, Japan). Quantitative real-time PCR was performed using amplified cDNA, gene-specific primers, and a SYBR Green dye kit (TaKaRa, Japan) using QuantStudio 3 (Thermo Fisher Scientific, United States). GAPDH was used as the housekeeping gene. The relative gene expression was calculated by the 2^–Δ^ ^Δ^ ^*Ct*^ method. Mitochondrial DNA content was quantified as described previously (Ulmer et al., 2018). Genomic and mitochondrial DNA was extracted with a genomic DNA extraction kit (BioFlux, China). The relative content of mtDNA was detected by quantitative real-time PCR using mitochondria-encoded NADH dehydrogenase (mt-ND1 or mt-ND2) primers and normalized to β-globin. All primers were purchased from TSINGKE Biological, and the sequences of the primers are listed in Supplementary Table S1.

### Protein Extraction and Western Blotting

Cells were lysed with a solution containing lysis buffer (KeyGEN, China), protease inhibitors, phosphatase inhibitors, and PMSF (Beyotime, China) at 4°C for 30 min on a rocker. A BCA protein assay kit (KeyGEN, China) was used to quantify total protein. Protein samples were mixed with 5× buffer and boiled for 10 min before loading onto a 10% SDS-PAGE gel (Epizyme, China). After electrophoresis, the proteins were transferred to polyvinylidene difluoride (PVDF) membranes (Millipore, United States). Based on the position of the markers, the membranes were cut into pieces, blocked with QuickBlock^TM^ blocking buffer (Beyotime, China) for 1 h, and incubated with primary antibodies (Supplementary Table S2) overnight at 4°C. The membranes were washed with TBST and then incubated with the corresponding secondary antibody (Supplementary Table S2) at room temperature for 1 h. The membranes were developed by ECL (Millipore, United States) using a ChemiDoc^TM^ Touch imaging system (Bio-Rad, United States). Intensity of the Western blot bands was quantified using Quantity One (United States).

### ATP Content Detection

Cellular ATP content was measured by an ATP assay kit (Beyotime, China) according to the manufacturer’s instructions. Cell lysates were centrifuged at 12,000 rpm for 5 min at 4°C, and the supernatant was used for subsequent determination. ATP detection working solution was added into the detection wells; sample or standard was added and quickly mixed with a pipettor, and the chemiluminescence signal (RLU) was measured with a chemiluminescence instrument (BioTek, United States) after at least a 2 s interval. Concentrations of ATP in the samples were calculated according to the standard curve; total protein concentration in the sample was determined by a BCA protein assay kit, and ATP concentration was converted into nmol/mg protein.

### HK Activities and Lactate Production Assays

hiPSC-CMs were seeded into 12-well plates at a density of 1 × 10^6^ cells and treated with 0.5 mM AICAR (Selleck, United States) or vehicle (DMSO) for 7 days from day 23 to day 30. Cellular HK activities and lactate production were measured by HK assay kit (Solarbio^®^ BC0745, China) and LA assay kit (Solarbi^®^ oBC2230, China) according to the manufacturer’s instructions, respectively. All experiments were normalized by the cell numbers.

### Immunofluorescence Staining

Cells were dissociated from 12-well plates and replated into Matrigel-coated coverslips. Cells were washed with PBS and fixed in 4% paraformaldehyde or 75% ice-cold ethanol for 20 min. Then, the cells were permeabilized with 0.5% Triton X-100 for 15 min, blocked with 5% BSA for 1 h, and incubated with primary antibodies (Supplementary Table S2) overnight at 4°C. After washing with PBS, the cells were incubated with secondary antibodies (Supplementary Table S2) for 1 h at room temperature in the dark and then stained using Hoechst 33342 (Beyotime, China) for 10 min. Imaging was performed using a fluorescence microscope (BX51; Olympus) and quantified using NIS-Elements software. Cells were analyzed for cell area, perimeter, circularity index, and sarcomere length with ImageJ software (United States). The circularity index equals 4Π^∗^Area/Perimeter^2^, and the sarcomere length was measured in two adjacent α-actinin bands (Liu et al., 2020).

### Transmission Electron Microscopy

The cells were fixed with 4% glutaraldehyde solution for 2 h and postfixed for 2 h with 1% osmium tetroxide at 4°C. The cells were dehydrated with ethanol and methanol gradients and then embedded in epoxy resin. The samples were cut into thin (60 nm) sections and stained with citrate and uranyl acetate. TEM images were acquired at random locations throughout the samples and captured using a transmission electron microscope (TEM; H-7500).

### Seahorse XF24 Metabolic Flux Analysis

The OCR was detected using a Seahorse XF24 extracellular flux analyzer (Agilent Technologies, United States). hiPSCs were cultured in a Matrigel-coated Seahorse XF-24 cell plate before analysis. On day 23, hiPSC-CMs were dissociated and subsequently plated onto a Seahorse XF-24 cell plate at 2.5 × 10^5^ cells/well. hiPSC-CMs were treated with 0.5 mM AICAR (Selleck, United States) or vehicle (DMSO) for 7 days. Mitochondrial function was analyzed using an XF Cell Mito Stress kit (Agilent Technologies, United States). Cells were washed with XF assay medium (unbuffered DMEM + 10 mM glucose + 2 mM L-glutamine + 1 mM sodium pyruvate) 1 h before the assay and incubated in 500 μL of the base medium in a non-CO_2_ incubator at 37°C. OCRs were analyzed by sequential automatic injections of mitochondrial inhibitors, including 2.5 μM oligomycin, 2 μM FCCP, 0.5 μM rotenone, and 0.5 μM antimycin A. OCR (pmol/min) was measured according to the manufacturer’s instructions (Agilent Technologies, United States). The results were used to calculate basal respiration, maximal respiration, proton leakage, ATP production, and non-mitochondrial respiration. The results were normalized to 1 μg of protein determined by a BCA protein assay kit.

Fatty acid oxidation (FAO) was assessed by an Agilent Seahorse XF Substrate Oxidation Stress Test Kit (Agilent Technologies, United States), same with mitochondrial stress test. Except for OCR assessment, there are four reagents Etomoxir (Eto, 4 μM), oligomycin [1.5 μM), FCCP (2 μM), and antimycin A/rotenone (0.5 μM)] were added to the system, other operation of the experiment is similar to Mito Stress Test. Basal respiration, proton leakage, maximal respiration, ATP product and spare respiratory capacity were measured in a XF24 analyzer.

### Mitochondrial Morphology Analysis

Mitochondrial morphology was visualized using Mito-Tracker^®^, green (Beyotime, China) according to the manufacturer’s instructions. Cells were incubated with 25 nM MitoTracker green for 30 min at 37°C. Then, the cells were washed with PBS and imaged utilizing a confocal microscope (Nikon, Japan). The average fluorescence intensity was analyzed by NIS-Elements Viewer software (Japan).

### Mitochondrial Membrane Potential Assay

Mitochondrial membrane potential (ΔΨm) was measured utilizing a mitochondrial membrane potential assay kit with JC-1 (Beyotime, China) according to the manufacturer’s instructions. Cells were incubated with JC-1 working solution for 30 min at 37°C. Then, the nuclei were stained with Hoechst 33342 (Beyotime, China) for 10 min at 37°C. Then, the cells were washed with PBS and imaged utilizing a confocal microscope (Nikon, Japan). At low ΔΨm, JC-1 is a green fluorescent monomer. At higher ΔΨm, JC-1 forms red fluorescent aggregates. The average fluorescence intensity was analyzed by NIS-Elements Viewer software. Data are shown as a ratio of red fluorescence intensity to green fluorescence intensity.

### Cell Counting Kit-8 (CCK-8) Assay

hiPSCs were cultured in a 96-well plate and then treated with various concentrations of AICAR for 7 days. CCK-8 solution (Solarbio, China) was added to each medium, and the conditions were maintained for 3 h at 37°C. Optical density was measured by a plate reader (BioTek, United States) at a wavelength of 450 nm. Every measurement was performed at least three times.

### Flow Cytometry

hiPSC-CMs were dissociated into single cells, washed with sterile DPBS and pelleted at 200 × g for 5 min. Following fixation in 4% paraformaldehyde, the cells were washed twice with DPBS, pelleted and resuspended in 200 μl of DPBS. Fixed hiPSCs and hiPSC-CMs were incubated with fluorescently labeled anti-cTnT antibodies (see Supplementary Table S1) for 1 h at room temperature, respectively. The cells were then washed three times. Analysis was performed on BD FACSCanto II cytometer, and the results were analyzed and plotted using FlowJo v10 software.

### Statistics

The data are expressed as the mean and standard error of the mean (SEM). Each measurement was performed at least three times. *P*-values were evaluated by unpaired t-test or one-way ANOVA using GraphPad Prism software (United States). For all analyses, a value of *P* < 0.05 was considered significant.

## Results

### Characterization of hiPSCs and hiPSC-CMs

hiPSCs cultured on Matrigel-coated plates formed colonies with tight arrangement and clear boundaries according to microscopy (Supplementary Figure S1B). hiPSCs consistently stained positive for the pluripotent stem cell-specific markers Nanog and Sox2 (Supplementary Figure S1D). Then, hiPSCs were differentiated into cardiomyocytes using a monolayer protocol according to the cardiac differentiation method (Supplementary Figure S1A). hiPSC-CMs began spontaneous contraction after 8 days of differentiation. hiPSC-CMs were purified on day 14, digested, and replated on day 20. The cells grew in a monolayer and showed a clear cell shape on Matrigel-coated plates (Supplementary Figure S1D). The flow analysis indicated that metabolic purification led to a purified population containing more than 92% cTnT-positive cells by day 20 (Supplementary Figure S1C). Immunostaining demonstrated that hiPSC-derived CMs expressed CM-specific markers, sarcomeric α-actinin, Cx43, and cTnT (Supplementary Figure S1E). Immunofluorescence detection of MLC2v, and MLC2a (Supplementary Figure S1F) showed that hiPSC-CMs mainly consist of ventricular CMs and a few other atrial CMs.

### Changes in Mitochondrial Morphology and Respiratory Function After the Differentiation of hiPSCs Into hiPSC-CMs

**FIGURE 1:**
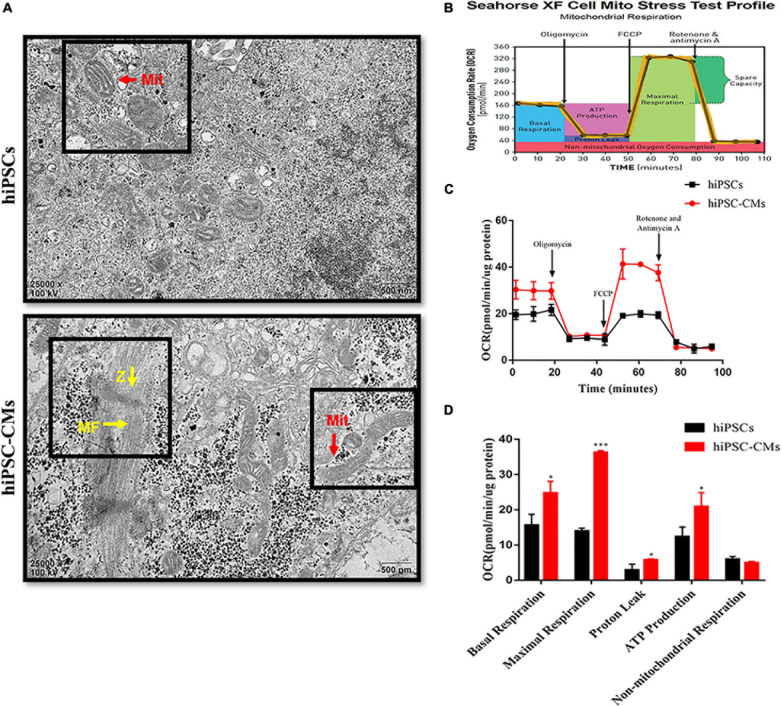
Changes in mitochondrial morphology and respiratory function after differentiation of hiPSCs into hiPSC-derived CMs. **(A)** Transmission electron microscopy images of hiPSCs and hiPSC-CMs illustrating the differences in mitochondrial morphology. Scale bar = 500 nm. hiPSC-CMs showed more mature, elongated, and large mitochondria with denser intramitochondrial cristae compared to those in hiPSCs. hiPSC-CMs had visible Z-bands and regular arrangement of myofibrils. Z, Z-bands, yellow arrows; MF, myofibrils; yellow arrows; Mit, mitochondria, red arrows. **(B)** Scheme of the mitochondrial stress test in the cells. **(C)** Two representative OCR traces of hiPSCs and hiPSC-CMs in response to oligomycin, FCCP, rotenone, and antimycin A (*n* = 12). **(D)** Statistical analysis of the differences in basal respiration, maximal respiration, proton leakage, ATP production, and non-mitochondrial respiration (*n* = 12). *P < 0.05, ***P < 0.001, compared with hiPSCs, unpaired *t*-test.

The ultrastructural changes in mitochondria in hiPSCs and hiPSC-derived CMs were analyzed by transmission electron microscopy. Images of hiPSCs suggest that the mitochondria were granular with sparse cristae (Figure 1A). We found that the mitochondria were characterized by the presence of a few more mature, elongated, and large mitochondria with denser intramitochondrial cristae and a compacted matrix in hiPSC-CMs compared to those in hiPSCs. We also observed that hiPSC-CMs contain Z-bands and regular arrangement of myofibrils (Figure 1A). These findings are consistent with the data of previous studies on myocardial structure during cardiomyocyte differentiation (Hoque et al., 2018). The density of mitochondria in cardiomyocytes is high, which contributes to ATP production (Piquereau et al., 2013). To determine the metabolic status of hiPSCs and hiPSC-CMs, we measured the OCR of the cells by an XF24 extracellular flux analyzer, which reflects the cellular respiration capacity. The detection diagram is shown in Figure 1B. We observed that hiPSC-CMs had significantly higher OCRs associated with basal respiration, ATP production, maximal respiration, and proton leakage compared to those in hiPSCs (Figures 1C,D). An increase in these mitochondrial oxidation parameters indicated the maturation or enhancement of mitochondrial oxidative capacity.

### The Change of AMPK Activation During Cardiomyocyte Differentiation of hiPSCs

**FIGURE 2:**
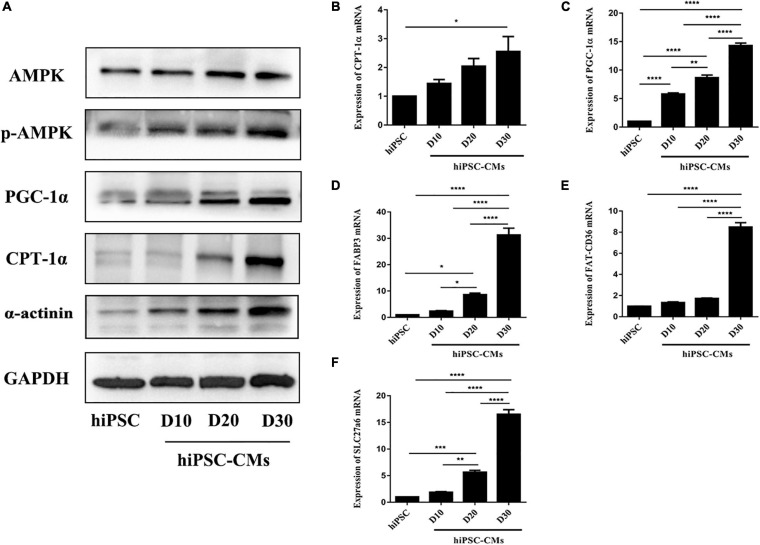
Activation of AMPK during hiPSC-derived cardiomyocyte differentiation. **(A)** Western blot analysis of AMPK, phospho-AMPK, PGC-1α, CPT-1α, and α-actinin on days 0, 10, 20, and 30 during hiPSC-derived cardiomyocyte differentiation. RT-qPCR analysis of the genes regulating energy metabolism [PGC-1α **(B)** and CPT-1α **(C)**] and fatty acid transport and oxidation-related genes [FABP3 **(D)**, FAT-CD36 **(E),** and SLC27a6 **(F)**] in hiPSC-derived cardiomyocytes on days 0, 10, 20, and 30 (*n* = 3). *P < 0.05, **P < 0.01, ***P < 0.001, ****P < 0.0001 one-way ANOVA.

AMPK is an energy-sensing protein kinase that plays a key role in the regulation of cellular energy metabolism (Herzig and Shaw, 2018); however, the role of AMPK in the maturation of hiPSC-CMs has not been defined. We examined the expression profile of AMPK during cardiac maturation of hiPSCs. The phosphorylation of AMPK was time-dependently increased in hiPSC-CMs (Figure 2A). Simultaneously, the protein levels of the downstream genes PGC-1α and CPT1α, which regulate energy metabolism, were dramatically increased (Figure 2A). The protein level of α-actinin was upregulated during cardiac differentiation of hiPSCs (Figure 2A). Additionally, the expression of PGC-1α (a crucial regulator of oxidative metabolism in cardiac development) and CPT1α (a regulator of fatty acid transport in the mitochondrial membrane) mRNAs was significantly time-dependently upregulated in the hiPSC-CM culture (Figures 2B,C). The mRNA expression levels of FABP3, FAT-CD36, and SLC27a6 were significantly increased during hiPSC-CM maturation to promote fatty acid transport and oxidation (Figures 2D–F). Hence, these data demonstrate that enhanced energy metabolism of hiPSC-CMs is related to AMPK phosphorylation and that activation of AMPK may be an effective approach to promote hiPSC-CM maturation.

### Improvement of the Metabolic Maturation of hiPSC-CMs by Activation of AMPK

**FIGURE 3:**
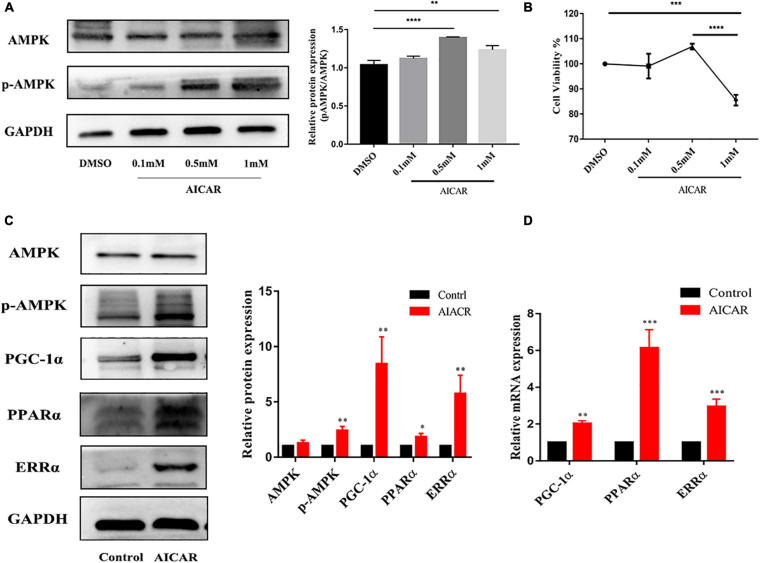
Activation of AMPK improved the metabolic maturation of hiPSC-CMs. **(A)** Western blot analysis of AMPK and phospho-AMPK expression in hiPSC-CMs treated with AICAR at various concentrations (0, 0.1, 0.5, and 1 mM). AICAR (0.5 and 1 mM) effectively upregulated the expression of phospho-AMPK (*n* = 3). **(B)** CCK-8 assay was performed to assess viability of hiPSC-CMs treated with AICAR at various concentrations (*n* = 3). **P < 0.01, ***P < 0.001, ****P < 0.0001 one-way ANOVA. **(C)** Western blot analysis of the effect of AMPK activation by AICAR on the protein expression levels of AMPK, p-AMPK, PGC-1α, PPARα, and ERRα (*n* = 3). **(D)** RT-qPCR analysis of the effect of AMPK activation by AICAR on the mRNA expression levels of PGC-1α, PPARα, and ERRα (*n* = 3). *P < 0.05, **P < 0.01, ***P < 0.001 compared with the control group, unpaired *t*-test.

**FIGURE 4:**
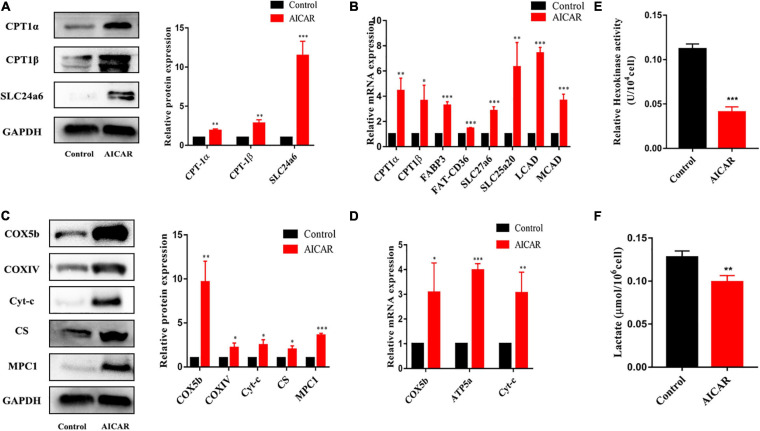
The effects of AMPK activation on the expression of the downstream metabolism-related targets. For **(A,C)**, the same membrane was probed for the indicated proteins, with GAPDH used as the loading control. **(A)** Western blot analysis of the expression of fatty acid β-oxidation-related proteins (CPT-1α, CPT-1β, and SLC24a6) in hiPSC-CMs treated with AICAR (vs. control) (*n* = 3). **(B)** RT-qPCR analysis of the effect of AICAR on the expression of fatty acid β-oxidation-related genes (CPT-1α, CPT-1β, FABP3, FAT-CD36, SLC27a6, SLC25a20, LCAD, and MCAD) (*n* = 3). **(C)** Western blot analysis of the expression of mitochondrial OxPhos-related proteins (COX5b, COXIV, Cyt-c, C-S, and MPC1) in hiPSC-CMs treated with AICAR (vs. control) (*n* = 3). **(D)** RT-qPCR analysis of the effect of AICAR on the expression of mitochondrial OxPhos-related genes (COX5b, ATP5a, and Cyt-c) (*n* = 3). **(E)** Relative hexokinase enzyme activity in hiPSCs treated with AICAR or control (*n* = 3). **(F)** Lactate measurements of hiPSCs treated with AICAR or control (*n* = 3). *P < 0.05, **P < 0.01, ***P < 0.001 compared with the control group, unpaired *t*-test.

To determine whether AMPK activation promotes energy metabolism and maturation of hiPSC-CMs, we treated hiPSC-CMs after replating for 7 days with AICAR (AMPK activator) at various concentrations (0, 0.1, 0.5, and 1 mM). Western blot was used to detect the protein expression of AMPK and phospho-AMPK, and CCK-8 assay was used to assess cell viability. As shown in Figures 3A,B, the activation effect of AICAR on AMPK was the strongest at a concentration of 0.5 mM and did not show significant cytotoxicity. This treatment condition was used in the following experiments. AMPK activation was reported to modulate glucose and fatty acid metabolism and mitochondrial function (Qi and Young, 2015). Therefore, hiPSC-CMs (day 23) were treated with DMSO (control) or AICAR at a concentration of 0.5 mM for 7 days and then subjected to biological and functional analysis on day 30. As shown in Figure 3C, AICAR increased the expression of phosphorylated AMPK and did not change the total AMPK protein levels. With regard to the effects of AMPK on the expression of the downstream metabolism-related targets, we found that AICAR increased the protein and transcript levels of PGC-1α, which improved mitochondrial biogenesis and metabolic maturation (Figures 3C,D; Liu et al., 2020). We observed significantly increased PPARα protein and mRNA levels in AICAR-treated cells compared to those in the control cells suggesting that AICAR treatment increased fatty acid β-oxidation (Figures 3C,D; Colasante et al., 2015). Additionally, AICAR treatment significantly increased the expression of ERRα (Figures 3C,D), which plays a key role in mitochondrial fatty acid oxidation (Alaynick, 2008). Western blot analysis revealed that the expression of fatty acid β-oxidation (CPT-1α, CPT-1β, and SLC24a6) and mitochondrial OxPhos markers (COX5b, COXIV, Cyt-c, C-S, and MPC1) was significantly upregulated in hiPSC-CMs following AICAR treatment compared to those in the control (Figures 4A,C). AICAR treatment significantly increased the mRNA expression of fatty acid β-oxidation (CPT-1α, CPT-1β, FABP3, FAT-CD36, SLC27a6, SLC25a20, LCAD, and MCAD) and mitochondrial OxPhos markers (COX5b, ATP5a, and Cyt-c) (Figures 4B,D).

We then used the same hexokinase assay to compare the glycolysis rates of hiPSC-CMs treated with AICAR or control. hiPSC-CMs treated with AICAR had significantly lower hexokinase activities compared with control group (Figure 4E). Glycolysis results in the conversion of glucose into pyruvate, which is subsequently reduced into lactate. To further validate that the increase in hexokinase activity is because of an increase in glycolysis, we compared the total cellular lactate levels in cardiomyocytes using a commercial lactate assay kit (Figure 4F). hiPSC-CMs treated with AICAR had significantly lower lactate levels compared with control group. hiPSC-CMs treated with AICAR had reduced glycolysis rates as would be expected during myocardial maturation.

### Improvement of the Morphological and Structural Maturation of hiPSC-CMs by Activation of AMPK

**FIGURE 5:**
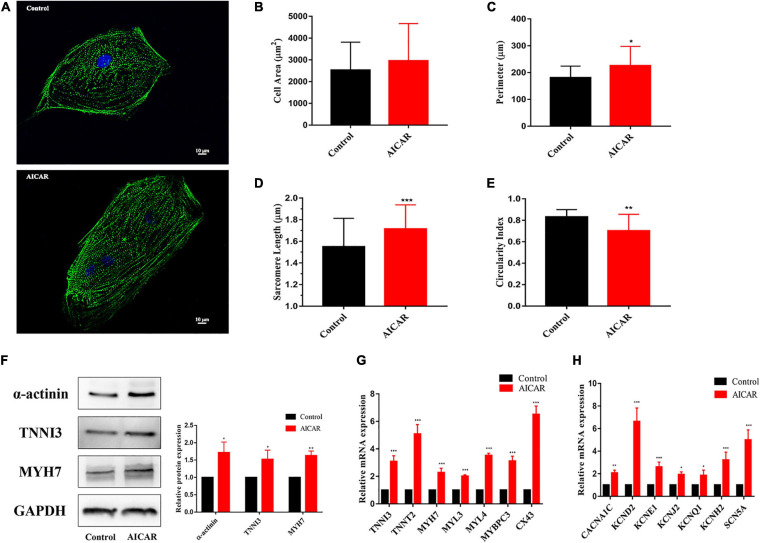
Activation of AMPK improves the morphological and structural maturation of hiPSC-CMs. **(A)** Representative immunostaining of α-actinin (green) and Hoechst 33342 (blue) in control or AICAR-treated hiPSC-CMs. Scale bar, 10 μm. Analysis of cell area **(B)**, perimeter **(C)**, sarcomere length **(D)**, and circularity index **(E)** (*n* = 80–100 cells/group). AICAR-treated hiPSC-CMs manifested significant changes in perimeter, sarcomere length and circularity index compared to those in the control cells. **(F)** Western blot analysis of α-actinin, TNNI3, and MYH7 protein expression in hiPSC-CMs treated with AICAR vs. control (*n* = 3). **(G)** RT-qPCR analysis of the effect of AICAR on the expression of the cardiomyocyte-related sarcomere protein-encoding gene (TNNI3, TNNT2, MYH7, MYL3, MYL4, MYBPC3, and CX43) (*n* = 3). **(H)** RT-qPCR analysis of the effect of AICAR on the expression of electrophysiology-related genes (CACNA1C, KCND2, KCNE1, KCNJ2, KCNQ1, KCNH2, and SCN5A) (*n* = 3). *P < 0.05, **P < 0.01, ***P < 0.001 compared with the control group, unpaired *t*-test.

To explore the effect of AICAR treatment on the morphology and gene expression of hiPSC-CMs, the cells with antibodies to α-actinin. In the AICAR treatment and control groups, the results of α-actinin immunostaining showed a clear sarcomere structure (Figure 5A). Additionally, AICAR-treated hiPSC-CMs had longer sarcomeres and lower circularity index than those in the control group (Figures 5B–E). Immunostaining demonstrated that In the AICAR treatment and control groups both expressed CM-specific markers TNNI3, and MYH7 (Supplementary Figures S2B,C).

We found that the protein levels of α-actinin, TNNI3, and MYH7 were significantly increased in AICAR-treated cells compared with that in the control cells (Figure 5F). We also analyzed the effect of AICAR treatment on the gene expression profiles of hiPSC-CMs. RT-PCR results showed that most CM-related sarcomere protein-encoding genes (TNNI3, TNNT2, MYH7, MYL3, MYL4, MYBPC3, and CX43) were significantly upregulated in AICAR-treated hiPSC-CMs (Figure 5G). Additionally, AICAR treatment induced the expression of calcium voltage-gated channel subunit (CACNA1C), potassium voltage-gated channel subfamily genes (KCND2, KCNE1, KCNJ2, KCNQ1, and KCNH2), and sodium voltage-gated channel subunit gene (SCN5A) (Figure 5H). These data suggest that AICAR treatment promotes the maturation process of hiPSC-CMs in multiple aspects, including cell morphology, sarcomeric structure, ultrastructural change, and gene expression.

### Improvement of Mitochondrial Maturation of hiPSC-CMs by Activation of AMPK

**FIGURE 6:**
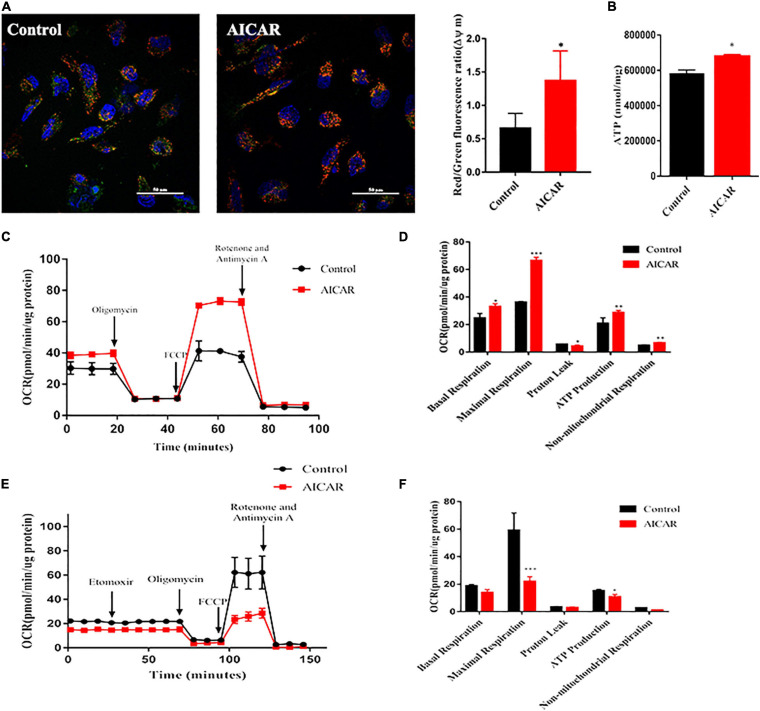
Activation of AMPK improves the functional maturation of mitochondria in hiPSC-CMs. **(A)** Mitochondrial membrane potential was measured using a fluorescence probe JC-1 assay system in hiPSC-CMs treated with AICAR vs. control. The ratio of red/green fluorescence represents the level of Δψm (*n* = 80–100 cells/group). Scale bar, 50 μm. **(B)** ATP levels of hiPSCs treated with AICAR or control (*n* = 3). **(C)** Two representative OCR traces of hiPSCs treated with AICAR or control in response to oligomycin, FCCP, rotenone, and antimycin A (*n* = 12). **(D)** Statistical analysis of the differences in basal respiration, maximal respiration, proton leakage, ATP production, and non-mitochondrial respiration (*n* = 12). **(E)** Representative fatty acid oxidation of hiPSCs treated with AICAR or control after incubation with the etomoxir, oligomycin, FCCP and rotenone, and antimycin A. **(F)** Statistical analysis of the differences in basal respiration, maximal respiration, proton leakage, ATP production, and non-mitochondrial respiration (*n* = 12). *P < 0.05, **P < 0.01, ***P < 0.001 compared with the control group, unpaired *t*-test.

The mitochondrion is an energy center of cardiomyocytes that controls cardiomyocyte metabolism and survival by constantly providing ATP. In the process of development, mitochondria have evolved in morphology and function (Yang et al., 2014b). We used JC-1 dye as an indicator of mitochondrial membrane potential (ΔΨm) to validate the changes in mitochondrial function. Results of confocal microscopy showed that AICAR treatment induced an overall increase in ΔΨm as compared to that in the control (Figure 6A). To characterize mitochondrial energy metabolism, we investigated the effect of AICAR treatment on mitochondrial respiration by the addition of oligomycin, FCCP, rotenone, and antimycin A (Figure 6C). AICAR treatment significantly increased the OCRs associated with basal respiration, maximal respiration, spare respiratory capacity, and ATP production (Figure 6D). Additionally, ATP production was increased by AICAR treatment of hiPSC-CMs (Figure 6B). In the Substrate Oxidation Stress Test, Etomoxir, a specific inhibitor of carnitine palmitoyl transferase 1α (CPT1α), was used to specifically inhibit mitochondrial FAO. Treatment with ETO abolished OCR increases in AICAR-treatment hiPSC-CMs. The increased fatty acid oxidation energy was shown by the decrease in OCR upon incubation with etomoxir in hiPSC-CMs treated with AICAR (Figure 6E). The maximum respiration rate, and the production of ATP were lower in the AICAR-treated hiPSC-CMs than that in the control group (Figure 6F). Thus, these data suggest that activation of AMPK may enhance mitochondrial oxidative capacity and promote metabolic maturation of hiPSC-CMs.

**FIGURE 7:**
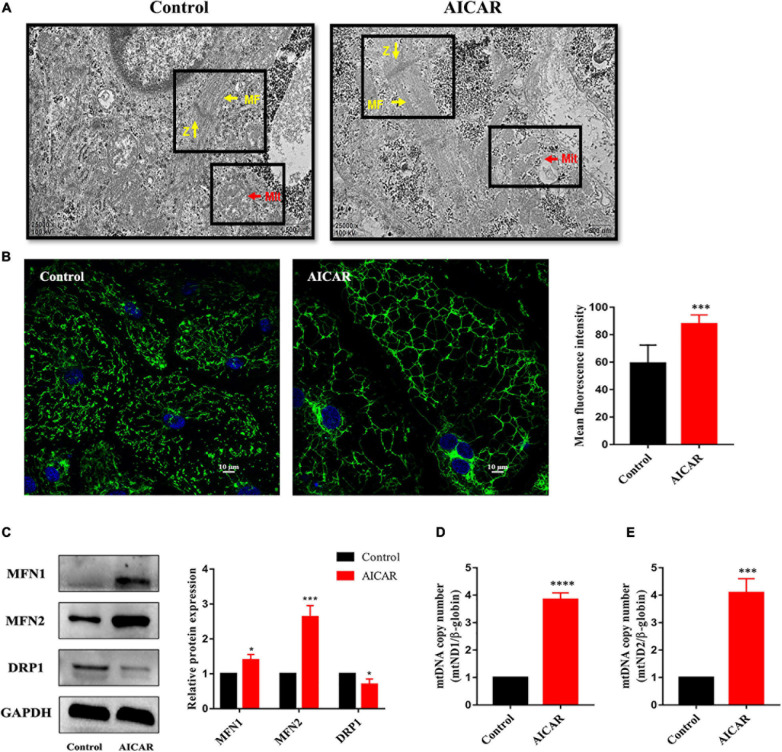
Activation of AMPK improves mitochondrial maturation of hiPSC-CMs. **(A)** Transmission electron microscopy images of hiPSCs treated with AICAR or control showing the differences in mitochondrial morphology. Scale bar, 500 nm. The number of mitochondria in AICAR-treated hiPSC-CMs was increased, and AICAR-treated hiPSC-CMs contained more mitochondrial crista than those in the control. Z, Z-bands, yellow arrows; MF, myofibrils, yellow arrows; Mit, mitochondria, red arrows. **(B)** MitoTracker green was used to investigate the changes in mitochondrial morphology in hiPSC-CMs treated with AICAR or control. The mean fluorescence intensity of MitoTracker green in hiPSC-CMs treated with AICAR vs. control (*n* = 80–100 cells/group). Scale bar, 10 μm. **(C)** Western blot analysis of MFN1, MFN2, and DRP1 protein expression in hiPSC-CMs treated with AICAR (vs. control) (*n* = 3). Mitochondrial DNA content determined by RT-qPCR using primers for mt-ND1 **(D)** and mt-ND2 **(E)** normalized to the housekeeping gene β-globin (*n* = 3). *P < 0.05, ***P < 0.001, ****P < 0.0001compared with the control group, unpaired *t*-test.

Then, hiPSC-CMs treated with AICAR or control were imaged using transmission electron microscopy to visualize the cellular ultrastructure and mitochondrial morphology. We observed that the number of mitochondria was increased in AICAR-treated hiPSC-CMs and that AICAR-treated hiPSC-CMs contained more mitochondrial crista than those in the control (Figure 7A). In control and AICAR-treated hiPSC-CMs, the myofibrils were plentiful and well-organized, and the Z-lines of the muscle fibers were visible (Figure 7A). Imaging of the cellular distribution of mitochondria using MitoTracker revealed disparate mitochondrial morphologies between control and AICAR-treated hiPSC-CMs; the former contained isolated mitochondria with fragments, and the latter consisting of an extensively interconnected filamentous network (Figure 7B). The mean fluorescence intensity of MitoTracker in AICAR-treated hiPSC-CMs was increased compared to that in the control (Figure 7B). Moreover, western blot analysis showed that mitochondrial fusion proteins MFN1 and MFN2 were upregulated, and a fission protein was downregulated compared with those in the control (Figure 7C). To determine whether AICAR treatment influenced the mitochondrial DNA content, we detected the mtDNA copy number by qPCR. AICAR treatment induced upregulation of the mitochondrial DNA to nuclear DNA ratio (Figures 7D,E). Overall, these data indicate that activation of AMPK increases mitochondrial fusion events leading to mitochondrial network expansion.

## Discussion

Induced pluripotent stem cell-derived cardiomyocytes (iPSC-CMs) can provide a good platform for alternative therapies for myocardial infarction, *in vitro* disease modeling, and drug toxicity testing (Wu et al., 2019). However, there are large differences between iPSC-CMs and mature cardiomyocytes in morphology, physiological function, metabolism, and gene expression (Ahmed et al., 2020). The developmental immaturity of hiPSC-CMs, difficulty of high-throughput evaluation of functional phenotypes, and inability to achieve effective reproduction of disease phenotypes may affect the accuracy and effectiveness of hiPSC-CMs in clinical applications. Therefore, a large number of studies have been investigating the methods to promote the maturation of hiPSC-CMs by using biochemical stimulation, physical stimulation, and 3D culture (Ahmed et al., 2020), including prolonged culture time (Kamakura et al., 2013), hormone treatment (Parikh et al., 2017), and electrical stimulation (Ronaldson-Bouchard et al., 2018). However, few studies have investigated associated molecular mechanisms in detail.

Cardiomyocytes require high metabolic activity. Metabolic activity is mainly maintained by the function of mitochondria, which is central to differentiation. Mitochondria undergo morphological changes during cell transition. Undifferentiated hiPSCs contain immature mitochondria, and their cristae are dysplastic and mainly located in the perinuclear region of the cytoplasm and the hypoplastic mitochondrial network (Jiang et al., 2018). When hiPSCs differentiate into cardiomyocytes, granular mitochondria become tubular and form a network of meshes, which contain elongated ridges that penetrate the cytoplasm (Hoque et al., 2018). Similar morphological changes during myocardial differentiation were detected in the present study. Consistent with the changes in mitochondrial morphology, cells have undergone significant metabolic changes that mainly include the enhancement of mitochondrial respiration. These results are consistent with the data of previous studies indicating that hiPSCs mainly produce ATP and maintain pluripotency through anaerobic glycolysis rather than mitochondrial OxPhos, as has been shown in hiPSC-CMS (Gu et al., 2016; Khacho and Slack, 2017). Additionally, our data indicated that phosphorylation of AMPK is time-dependently increased in hiPSC-CMs and that the expression of PGC-1α and CPT1α mRNA was significantly time-dependently upregulated in hiPSC-CMs. AMPK is energy-sensing protein kinase that plays a key role in the regulation of cellular energy metabolism. Several studies have shown that relatively mature hiPSC-CMs have highly phosphorylated AMPK (Yang et al., 2019; Sarikhani et al., 2020). AMPK has been shown to play a protective role in various cardiac pathophysiological processes to restore energy balance (Dolinsky and Dyck, 2006). AMPK can promote fat mobilization and release fatty acids from triglycerides by stimulating lipase, and free fatty acids are transported to the mitochondria for fatty acid β-oxidation (Dolinsky and Dyck, 2006). Moreover, AMPK can inactivate the phosphorylation of acetyl-Coenzyme A carboxylase 2 and reduce the inhibitory effect of malonyl CoA on carnitine acyltransferase 1 thereby promoting fatty acid oxidation (Herzig and Shaw, 2018). These reports confirm our results that activation of AMPK promotes fatty acid oxidation in hiPSC-CMs by inducing the expression of CPT-1, fatty acid transport protein (FATP), and fatty acid-binding protein (FABP).

Mitochondria are the site of oxidative phosphorylation (OxPhos). Increasing evidence shows that AMPK has specific regulatory effects on various aspects of mitochondrial biology and homeostasis (Herzig and Shaw, 2018). These effects include control of the number of mitochondria by stimulation of mitochondrial biogenesis, regulation of the shape of mitochondrial network, and control of the quality of mitochondria via autophagy and mitosis. Biogenesis of mitochondria is a response to increased energy consumption to produce more ATP (Herzig and Shaw, 2018). This observation is supported by an increase in mitochondrial biogenesis caused by continuous activation of AMPK (Bergeron et al., 2001). We used AICAR to induce AMPK activation in hiPSC-CMs. Our data indicate that AICAR significantly increased the OCR associated with basal respiration, ATP production, maximum respiration, and reserve capacity similar to an increase in the oxidative capacity of mature mitochondria indicating that cardiomyocytes were in a more mature metabolic state. These observations are consistent with recent reports (Rana et al., 2012; Correia et al., 2017). An increase in oxidative capacity may be related to an increase in the number of mitochondria, improvement of mitochondrial morphology (for example, enhancement of cristae development) and/or activity of the electron transport chain. An increase in the mtDNA/nDNA ratio also supports this possibility. The results of transmission electron microscopy showed that AICAR-treated hiPSC-CMs contain more mitochondria and manifest better mitochondrial morphology and mitochondrial content than those in the control. Our data indicate that activation of AMPK promotes mitochondrial fusion in hiPSC-CMs. When cells are under stress, mitochondrial respiratory reserve capacity is used to support an increase in energy demand thus assisting in the maintenance of cell and organ functions, cell repair, or detoxification of active substances (Hill et al., 2009). In hiPSC-CMs, AICAR treatment increased respiratory reserve capacity suggesting that the treated cells may perform better in the case of increased energy demand. Although AICAR-treated hiPSC-CMs are significantly more mature than control cells, it is important to recognize that the improvement in maturity is not complete. Therefore, changes in mitochondrial content are the key components of cardiomyocyte maturation during development (Yang et al., 2014a) and may contribute to an overall increase in mitochondrial oxidative phosphorylation. These results suggest that AMPK activation induces the expression of the downstream genes and promotes biogenesis and metabolic maturation of the mitochondria.

Previous studies have shown that hiPSCs can differentiate into cells with typical structural and functional characteristics of fetal cardiomyocytes; however, these cells are not sufficiently mature to be used as adult cardiomyocytes. Cardiomyocytes have different morphological characteristics at different stages. Mature cardiomyocytes have a large aspect ratio and are slender and rod-shaped, while hiPSC-CMs are mostly round (Wu et al., 2019). Our results showed that AICAR-treated hiPSC-CMs had an increased cell perimeter, decreased circularity index, and longer sarcomeres compared with those in the control group. The electron microscopy results showed that AICAR-treated hiPSC-CMs had more structured myofibrils. AICAR treatment changed cell morphology to more slender characteristics that are closer to that of adult cardiomyocytes. Considering that total differentiation and maturation time in our experiment was approximately 1 month, AICAR treatment may be a step forward in the timely maturation of hiPSC-CMs. Furthermore, hiPSC-CMs had low expression levels of important structural genes, such as MYH7, cTNI, and sarcoplasmic reticulum ATPase (Denning et al., 2016). The results of the present study demonstrated that AICAR treatment upregulated the expression of cardiac maturation-related genes. In general, our observations in combination with previous reports suggest that AICAR treatment can promote the maturation of hiPSC-CMs in cell morphology and gene expression (Drawnel et al., 2014).

In hiPSC-CMs, activation of AMPK induces a number of target genes that may promote maturation. Importantly, AICAR-treated hiPSC-CMs manifested a more mature phenotype than that in the control group in terms of mitochondrial oxidative metabolism, cell structure, and gene expression. Studies have reported that the maturation of CMs is characterized by structural, electrophysiological, and metabolic alterations together facilitating more efficient and increased function (Mills and Hudson, 2019). It is very important to maintain the activity and normal electrophysiological characteristics of differentiated cells. Electrophysiological characteristics are the manifestation of cell function. Although we have demonstrated that activation of AMPK by AICAR is an effective means to promote morphological and metabolic maturation of hiPSC-CMs, the effect of AMPK on electrophysiological maturation of hiPSC-CMs remain unclear. The limitation of this study is the failure to detect the changes of electrophysiological function. We will focus on the study of electrophysiological function in the future. For all that, activation of AMPK induces a number of target genes that may promote maturation in hiPSC-CMs. Importantly, AICAR-treated hiPSC-CMs manifested a more mature phenotype than that in the control group in terms of mitochondrial oxidative metabolism, cell structure, and gene expression. This study provides a foundation for subsequent clinical application of hiPSC-CMs and detailed investigation of cardiometabolic and regulatory mechanisms.

## Data Availability Statement

The raw data supporting the conclusions of this article will be made available by the authors, without undue reservation.

## Author Contributions

JZ and JT contributed to the conception and design of the study. LYe carried out the experiments and drafted the manuscript. BT contributed to edit the figures. QY and HX organized the database. LYa and QZ carried out the sample collection. YZ, MX, and XZ contributed to the cells culture, reagent procurement, and management. All authors contributed to manuscript revision, read, and approved the submitted version.

## Conflict of Interest

The authors declare that the research was conducted in the absence of any commercial or financial relationships that could be construed as a potential conflict of interest.

## Supplementary Material

Supplementary Figure 1 Characterization of human induced pluripotent stem cells (hiPSCs) and hiPSC-derived cardiomyocytes (hiPSC-CMs). **(A)** Scheme of the process of hiPSCs differentiation into cardiomyocytes. **(B)** Microscopic image showed hiPSCs growth as colonies (a). Microscopic image showing hiPSC-CMs growth as a monolayer (b). **(C)** The efficiency of cardiac differentiation after metabolic purification was 92.8% measured by flow cytometry for cTnT positive on day 20 cells. **(D)** Immunofluorescence staining indicated that hiPSCs express the pluripotent stem cell-specific markers Nanog (green) and Sox2 (green). Nuclei were stained with DAPI (blue). Scale bar, 50 μm. **(E)** Immunofluorescence staining indicated that hiPSC-CMs express the cardiomyocyte-specific markers α-actinin (green), Cx43 (green), and cTnT (red). Nuclei are stained with DAPI (blue). Scale bar, 20 μm. **(F)** Immunofluorescence staining indicated that hiPSC-CMs express the ventricular CMs-specific markers MLC2v (red), and atrial CMs-specific markers MLC2a (green). Nuclei were stained with DAPI (blue). Scale bar, 100 μm.

Supplementary Figure 2 **(A)** Immunofluorescence staining indicates that hiPSC-CMs express the cardiomyocyte-specific markers α-actinin (green). Nuclei were stained with DAPI (blue). Scale bar, 20 μm. **(B)** Immunofluorescence staining indicates that hiPSC-CMs express the cardiomyocyte-specific markers TNNI3 (green). Nuclei were stained with DAPI (blue). Scale bar, 50 μm. **(C)** Immunofluorescence staining indicates that hiPSC-CMs express the cardiomyocyte-specific markers MYH7 (red). Nuclei were stained with DAPI (blue). Scale bar, 20 μm. **(D)** MitoTracker green was used to investigate the changes in mitochondrial morphology in hiPSC-CMs treated with AICAR or control. Nuclei were stained with DAPI (blue). Scale bar, 20 μm.

Supplementary Table 1 Primer sequences for Quantitative Real-time PCR.

Supplementary Table 2 The antibodies used for Immunofluorescence and Western blot.

Supplementary Video 1 hiPSC-CMs began spontaneous contraction after 8 days of differentiation (day 8).

Supplementary Video 2 hiPSC-CMs were purified, digested, and replated. hiPSC-CMs began rhythmic and steady contraction (day 23).
