# Supplementary figures and images for: Activation of AMPK Promotes Maturation of Cardiomyocytes Derived From Human Induced Pluripotent Stem Cells
Source: Front Cell Dev Biol. 2021 Mar 9;9:644667. doi: 10.3389/fcell.2021.644667 (PMC7985185; doi:10.3389/fcell.2021.644667)

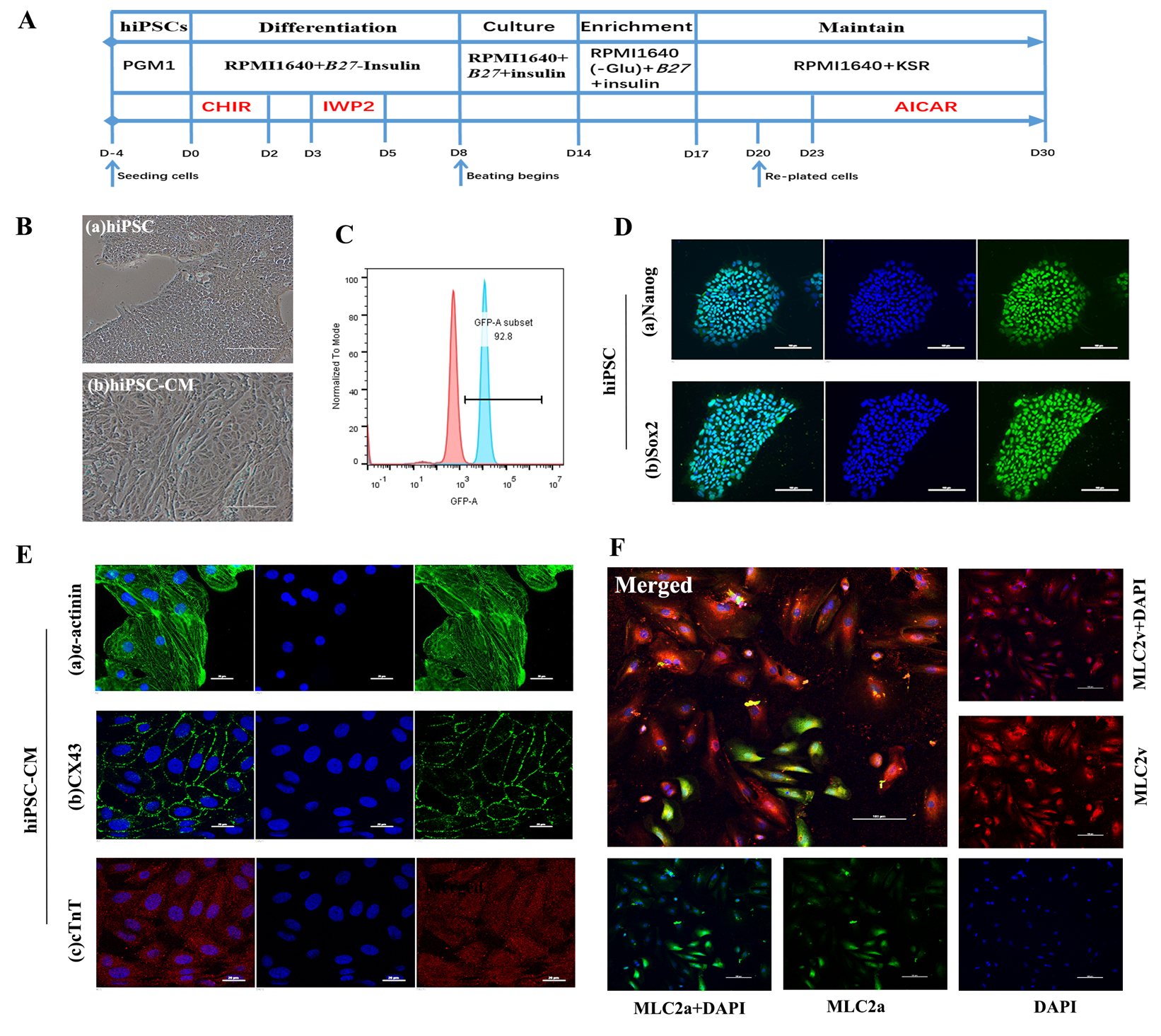

Supplement: Supplementary Figure 1 — Characterization of human induced pluripotent stem cells (hiPSCs) and hiPSC-derived cardiomyocytes (hiPSC-CMs). (A) Scheme of the process of hiPSCs differentiation into cardiomyocytes. (B) Microscopic image showed hiPSCs growth as colonies (a). Microscopic image showing hiPSC-CMs growth as a monolayer (b). (C) The efficiency of cardiac differentiation after metabolic purification was 92.8% measured by flow cytometry for cTnT positive on day 20 cells. (D) Immunofluorescence staining indicated that hiPSCs express the pluripotent stem cell-specific markers Nanog (green) and Sox2 (green). Nuclei were stained with DAPI (blue). Scale bar, 50 μm. (E) Immunofluorescence staining indicated that hiPSC-CMs express the cardiomyocyte-specific markers α-actinin (green), Cx43 (green), and cTnT (red). Nuclei are stained with DAPI (blue). Scale bar, 20 μm. (F) Immunofluorescence staining indicated that hiPSC-CMs express the ventricular CMs-specific markers MLC2v (red), and atrial CMs-specific markers MLC2a (green). Nuclei were stained with DAPI (blue). Scale bar, 100 μm. [file Image_1.TIF]

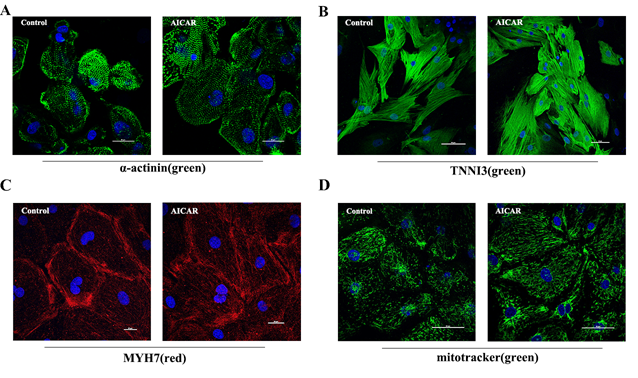

Supplement: Supplementary Figure 2 — (A) Immunofluorescence staining indicates that hiPSC-CMs express the cardiomyocyte-specific markers α-actinin (green). Nuclei were stained with DAPI (blue). Scale bar, 20 μm. (B) Immunofluorescence staining indicates that hiPSC-CMs express the cardiomyocyte-specific markers TNNI3 (green). Nuclei were stained with DAPI (blue). Scale bar, 50 μm. (C) Immunofluorescence staining indicates that hiPSC-CMs express the cardiomyocyte-specific markers MYH7 (red). Nuclei were stained with DAPI (blue). Scale bar, 20 μm. (D) MitoTracker green was used to investigate the changes in mitochondrial morphology in hiPSC-CMs treated with AICAR or control. Nuclei were stained with DAPI (blue). Scale bar, 20 μm. [file Image_2.TIF]
