# Supplementary material for: Activation of AMPK Promotes Maturation of Cardiomyocytes Derived From Human Induced Pluripotent Stem Cells
Source: Front Cell Dev Biol. 2021 Mar 9;9:644667. doi: 10.3389/fcell.2021.644667 (PMC7985185; doi:10.3389/fcell.2021.644667)
Supplement: Supplementary Table 1 — Primer sequences for Quantitative Real-time PCR. [file Table_1.docx]

**Supplemental Tables**

**Table S1.** Primer sequences for Quantitative Real-time PCR.

| **Gene** | Forward Primer Sequence (**5'to3'**) | | Reverse Primer Sequence **(5'to3')** |
| --- | --- | --- | --- |
| PGC-1α | GCTTTCTGGGTGGACTCAAG | | GAGGGCAATCCGTCTTCATCC |
| CPT-1α | CCTCAACGCTGAACACTCCT | | CCTCAACGCTGAACACTCCT |
| PPARα | AAGCTGTCACCACAGTAGCTTG | | AACGAATCGCGTTGTGTGAC |
| ERRα | AGGGTTCCTCTCGGAGACAGAG | | TCACAGGATGCCACACCATAG |
| FAT-CD36 | GCAACAAACCACACACTGGG | | AGACTGTGTTGTCCTCAGCG |
| FABP3 | GATGACAGGAAGGTCAAGTCCA | | GGTTTTGCCGCTTTAGCCTT |
| SLC27a6 | TTGAGTTGGGTGCCACTTGT | | TTCCAATTGCCAAACGCACC |
| CPT1β | CCTGGTGCTCAAGTCATGGT | | TGGATGATGTTTCCCAGGCG |
| COX5b | TGTGAAGAGGACAATACCAGC | | CCAGCTTGTAATGGGCTCC |
| Cyt-c | CTTTGGGCGGAAGACAGGTC | | TTATTGGCGGCTGTGTAAGAG |
| MCAD | ACAGGGGTTCAGACTGCTATT | | TCCTCCGTTGGTTATCCACAT |
| LCAD | AGGGGATCTGTACTCCGCAG | | CTCTGTCATTGCTATTGCACCA |
| SLC25a20 | GACCAGCCAAAACCCATCAG | | AGAGGGTGACCGACGAACA |
| ATP5a | GTATTGCCCGCGTACATGG | | AGGACATACCCTTTAAGCCTGA |
| ACOX1 | ACTCGCAGCCAGCGTTATG | | AGGGTCAGCGATGCCAAAC |
| TNNI3 | CCTCAAGCAGGTGAAGAAGG | | CAGTAGGCAGGAAGGCTCAG |
| TNNT2 | AGCATCTATAACTTGGAGGCAGAG | TGGAGACTTTCTGCTTATCGTTG | |
| MYL3 | GCCCTAAGGAGGTCGAGTTT | ACACTGCCCGTAGGTGATCT | |
| MYL4 | GACTTCACTGCCGACCAGAT | CTCGGCATTGGTAGGGTTCT | |
| MYH7 | GAGGACAAGGTCAACACCCT | CGCACCTTCTTCTCTTGCTC | |
| MYBPC3 | GGCATGCTAAAGAGGCTCAA | TCTTGTGGCCTTTGCTCAC | |
| CX43 | CAATCACTTGGCGTGACTTC | AAAGGCAGACTGCTCATCTC | |
| CACAC1C | GAAGCGGCAGCAATATGGGA | TTGGTGGCGTTGGAATCATCT | |
| SCN5A | TCTCTATGGCAATCCACCCCA | GAGGACATACAAGGCGTTGGT | |
| KCNE1 | ATGATCCTGTCTAACACCACAGC | GAATGGGTCGTTCGAGTGCT | |
| KCNJ2 | GTGCGAACCAACCGCTACA | CCAGCGAATGTCCACACAC | |
| KCNH2 | CACCGCCCTGTACTTCATCT | AGGCCTTGCATACAGGTTCA | |
| KCNQ1 | CGCCTGAACCGAGTAGAAGA | TGAAGCATGTCGGTGATGAG | |
| KCND2 | CTACCTGTTCCGGTGATTGTATCC | TCTTTTGTGCCCTTCGTTTGT | |
| GAPDH | GAAATCCCATCACCATCTTCCAG | AAATGAGCCCCAGCCTTCTC | |
| mt-ND1 | CATTCCTAATGCTTACCGAACGA | TGAGAGCTAAGGTCGGGGC | |
| mt-ND2 | CAGCACCACGACCCTACTAC | GCAGGGTGATGGTGGCTATG | |
| β-globin | GTGCTCGGTGCCTTTAGTGA | GGGGAAAGAAAACATCAAGCGT | |
