# Supplementary material for: Activation of AMPK Promotes Maturation of Cardiomyocytes Derived From Human Induced Pluripotent Stem Cells
Source: Front Cell Dev Biol. 2021 Mar 9;9:644667. doi: 10.3389/fcell.2021.644667 (PMC7985185; doi:10.3389/fcell.2021.644667)
Supplement: Supplementary Table 2 — The antibodies used for Immunofluorescence and Western blot. [file Table_2.docx]

**Supplemental Tables**

**Table S2.** The antibodies used for Immunofluorescence and Western blot.

| **Antibodies** | **Manufacturer** | **Assay** | **Cat. No.** |
| --- | --- | --- | --- |
| Goat anti-Rabbit IgG (H+L),FITC | ZSGB-BIO | IF | ZF-0311 |
| Goat anti-Rabbit IgG (H+L),Alexa Fluor® 488 | ZSGB-BIO | IF | ZF-0511 |
| SOX2 | proteintech | IF | 11064-1-AP |
| Nanog | proteintech | IF | 14295-1-AP |
| cTnT | Abcam | IF | ab10214 |
| Cx43 | Abcam | IF | ab11370 |
| α-actinin | proteintech | IF/WB | 11313-2-AP |
| AMPK | proteintech | WB | 10929-2-AP |
| Phospho-AMPK  (Thr 172) | Affinity | WB | AF3423 |
| CPT1α | proteintech | WB | 15184-1-AP |
| CPT1β | proteintech | WB | 22170-1-AP |
| TNNI3 | proteintech | WB | 21652-1-AP |
| MYH7 | proteintech | WB | 22280-1-AP |
| PPARα | Abcam | WB | ab227074 |
| ERRα | Abcam | WB | AB76228 |
| PGC-1α | Santa cruz biotechnology | WB | sc-518025 |
| MFN1 | Santa cruz biotechnology | WB | sc-166644 |
| MFN2 | Santa cruz biotechnology | WB | sc-515647 |
| DRP1 | proteintech | WB | 12957-1-AP |
| CS | Santa cruz biotechnology | WB | sc-390693 |
| COXⅣ | abcam | WB | ab202554 |
| COX5b | Santa cruz biotechnology | WB | sc-374416 |
| Cyt-c | Abcam | WB | ab13575 |
| MPC1 | Cell Signaling Technology | WB | 14462S |
| GAPDH | Affinity | WB | AF7021 |
| Goat anti-Rabbit IgG (H+L) | ZSGB-BIO | WB | ZB-2301 |
| Rabbit anti-Mouse IgG (H+L) | ZEN-BIO | WB | 701051 |
